# Adverse Events of Extracorporeal Ultrasound-Guided High Intensity Focused Ultrasound Therapy

**DOI:** 10.1371/journal.pone.0026110

**Published:** 2011-12-14

**Authors:** Tinghe Yu, Jun Luo

**Affiliations:** 1 Laboratory of Obstetrics and Gynecology, The Second Affiliated Hospital, Chongqing Medical University, Chongqing, China; 2 Key Laboratory of Obstetrics and Gynecology, Chongqing Bureau of Health, Chongqing, China; 3 Hospital of Stomatology, Chongqing Medical University, Chongqing, China; Cedars-Sinai Medical Center, United States of America

## Abstract

**Background:**

High-intensity focused ultrasound (HIFU) is considered to be an alternative to surgery. Extracorporeal ultrasound-guided HIFU (USgFU) has been clinically used to treat solid tumors. Preliminary trials in a small sample of a Western population suggested that this modality was safe. Most trials are performed in China thereby providing comprehensive data for understanding the safety profile. The aim of this study was to evaluate adverse events of USgFU therapy.

**Methods and Findings:**

Clinical data were searched in 2 Chinese databases. Adverse events of USgFU were summarized and compared with those of magnetic resonance-guided HIFU (MRgFU; for uterine, bone or breast tumor) and transrectal ultrasound-guided HIFU (for prostate cancer or benign prostate hyperplasia). USgFU treatment was performed using 7 types of device. Side effects were evaluated in 13262 cases. There were fewer adverse events in benign lesions than in malignant lesions (11.81% *vs.* 21.65%, p<0.0001). Rates of adverse events greatly varied between the disease types (0–280%, p<0.0001) and between the applied HIFU devices in both malignant (10.58–44.38%, p<0.0001) and benign lesions (1.67–17.57%, p<0.0001). Chronological analysis did not demonstrate a decrease in the rate of adverse events. Based upon evaluable adverse events, incidences in USgFU were consistent with those in MRgFU or transrectal HIFU. Some side effects frequently occurred following transrectal HIFU were not reported in USgFU. Several events including intrahepatic metastasis, intraoperative high fever, and occlusions of the superior mesenteric artery should be of particular concern because they have not been previously noted. The types of adverse events suggested that they were ultrasonic lesions.

**Conclusion:**

The frequency of adverse events depended on the location of the lesion and the type of HIFU device; however, side effects of USgFU were not yet understood. USgFU did not decrease the incidence of adverse events compared with MRgFU.

## Introduction

High intensity focused ultrasound (HIFU) is a noninvasive therapeutic modality against solid lesions that is guided by magnetic resonance (MRgFU) or ultrasound imaging. Moreover, HIFU can be used as an alternative to surgery. Because ultrasound provides a rapid imaging technique, it may be possible to monitor tissue responses in real time using ultrasound-guided HIFU, thereby decreasing untoward lesions [1], [2]. MRgFU has been approved by the FDA for the treatment of uterine fibroids. Furthermore, it has been preliminarily tested in bone and breast cancers [3], [4]. Transrectal ultrasound-guided HIFU for prostate cancer has been approved in Europe and clinical trials are currently on-going in many countries [5].

Extracorporeal ultrasound-guided HIFU (USgFU) was clinically introduced as a treatment for solid tumors in the late 1990 s [6]. USgFU therapy has been approved in China, and clinical trials for cancers of liver, kidney and pancreas are in underway in Europe and Asia. Preliminary trials for liver and kidney cancers in the United Kingdom demonstrated the safety of USgFU; moreover, in those trials, adverse events (AE) such as discomfort, skin toxicity and edema at the treatment site, and mild fever were transitory [7], [8]. The small number of cases involved limits the clinical implications. Complications in 79 cases of liver and 35 cases of pancreas cancer in a Korean clinical center were recently summarized. All patients had local skin reactions (redness, edema and pain). Severe AEs, such as rib/vertebra necrosis, hydrothorax, pancreatitis, biliary obstruction, and fistula formation did occur [9]. The data indicate that the safety profile of USgFU treatment is an important concern.

Most clinical trials of USgFU have been performed in China. Over 20000 patients with malignant or benign diseases have received this treatment, providing sufficient data to thoroughly document the prevalence of treatment-related AEs. However, those results are commonly published in Chinese and are unavailable for scientists outside China. Some data have been recently released in English, but these reports only described a few disease types in a few clinical centers, hardly reflecting the scope of the safety profile [10], [11].

AEs following USgFU were summarized in this study. Incidences of AEs following USgFU were compared with those following MRgFU (uterine, bone, and breast tumors) or transrectal HIFU (prostate cancer and benign prostate hyperplasia). The findings indicated that the rate of AEs drastically varied between disease types and between HIFU devices. Several events should be of particular concern, because they have not been previously noted.

## Methods

### Ethics statement

All clinical trials examined in the present report were approved by the appropriate Institutional Review Boards and all patients signed consent forms, both of which were stated in the original articles. Thus, approval for the present retrospective study by an Institutional Review Board was not needed.

### Searching clinical trials

Published clinical reports of USgFU were searched in 2 databases, the Chinese Scientific & Technical Periodicals Database (www.cqvip.com) and China National Knowledge Infrastructure (www.cnki.net), using the terms “high intensity focused ultrasound” or “focused ultrasound”. The inclusion criterion was that AEs were quantitatively described in the article. Local reactions at the treatment site (mild skin symptoms and tolerable pain) and mild fever were not considered, as they occurred in almost all cases [9], [10].

### Statistics

Data were processed with the statistics software SAS (SAS Inst., Cary, NC). Chi-square test was used and correct for multiple comparison using a bootstrap method. p<0.05 was considered significance.

For the statistical comparisons, references 3, 4 and 12 served as the control reports for MRgFU, and references 5, 13 and 14 as the control reports for transrectal HIFU.

## Results

### General

686 articles involving 23601 patients with malignant/benign tumors and nontumorous diseases that occurred before December 2010 were identified in 2 databases. AEs were quantitatively described in 348 articles; thus only 13262 (56.19%) cases were included in the evaluation of side effects. HIFU treatments were performed using 7 kinds of devices (Table 1).

**Table 1:** Ultrasound-guided HIFU devices and the sample size in the clinical trial.

Device	Case (included/all)	Frequency (MHz)	Highest intensity (W/cm^2^)	Manufacturer
2000	94/363	1.0	≥1000	Shenzhen Xifukang Med. Treatment Technol. Co.
2001	775/1890	1.0	2000	Shanhai Jiaoda Shiye Co.
CZ-901	343/560	0.8	N/A	Mianyang Sonic Electronic
FEP-BY	6827/12139	0.8/1.0	4000	Beijing Yuande Biomed. Eng. Co.
HY2900	31/31	N/A	≥10000	Wuxi Haiying Electronic Med. System Co.
JC	2296/4005	0.8/0.9/1.0/1.6	20000	Chongqing Haifu Technol. Co.
NIT-9000	2896/4613	1.0	3000	Shanghai A&S Sci. Technol. Development Co.

Complications were multifarious. Incidences of AEs varied considerably between the disease types (0–280%, p<0.0001), and the rate in benign lesions was less than that in malignant lesions (11.81% *vs.* 21.65%, p<0.0001) (Tables S1, S2, S3, S4, S5, S6, S7).

### AEs in malignant diseases

AEs associated with 6 common cancers were summarized in Table 2.

**Table 2:** Summary of AEs that occurred in the treatment of 6 malignant and 2 benign diseases with USgFU.

Disease	Case	Adverse event	Incidence	
*Malignant*				
Liver	2201	Skin burn 493	35.30% (777/2201)	
		Rib injury 7		
		Chest wall injury 2		
		Vertebra injury 4		
		Severe abdomen pain 39		
		ALT/AST elevation 81		
		Jaundice aggravation 2		
		Cholecystitis 5		
		Intrahepatic cholangiectasis 5		
		Gastroenteric dysfunction 22		
		Rupture of esophageal varices 1		
		Supraventricular tachycardia/palpitation 12		
		Hydropericardium 2		
		Hypertension 8		
		Bleeding/liquefaction 7		
		Tumor rupture 2		
		Intrahepatic metastasis 7		
		Lung embolism 2		
		Hydrothorax 57		
		Pneumonedema 1		
		Asthma 2		
		Hematuria 8		
		Creatine elevation 2		
		Renal failure 1		
		High fever 4		
		Death 1		
Pancreas	1717	Burn 51	8.74% (150/1717)	
		Vertebra burn 2		
		Diabetes 22		
		Jaundice aggravation 10		
		Pancreatitis 32		
		Steatorrhea 13		
		Gastroenteric dysfunction 13		
		Bleeding 2		
		Occlusion of the superior mesenteric artery 1		
		Collapse 3		
		Hepatic abscess 1		
Bone	224	Skin burn 10	20.54% (46/224)	
		Nerve injury 14		
		Infection 2		
		Fracture 10		
		Epiphyseal separation 1		
		ALP elevation 5		
		Hemoglobinuria 1		
		Tumor rupture 2		
		Death 1		
Breast	167	Skin burn 19	11.38% (19/167)	
Soft tissues	81	Skin burn 5	14.81% (12/81)	
		Cutaneous necrosis 4		
		Nerve injury 3		
Prostate	375	Skin burn 27	28.53% (107/375)	
		Hematuria 65		
		Urinary obstruction 9		
		Urethral stricture 4		
		Incontinence 2		
*Benign*				
Uterine fibroid	5526	Skin burn 112	10.19% (563/5526)	
		Vertebra burn 23		
		Nerve injury 169		
		Severe/prolonged abdomen pain 92		
		Hematuria 159		
		Urinary irritation 1		
		Hemafecia 1		
		Gastroenteric dysfunction 6		
Prostate hyperplasia	883	Hematuria 157	24.80% (219/883)	
		Urinary irritation 38		
		Urine retention 24		

ALT: alanine aminotransferase, AST: aspartate aminotransferase, ALP: alkaline phosphatase.

*Liver*. 777 AEs (26 types) were observed in 2201 cases (35.30%). The most frequent event was a burn (22.99%), which occurred in both the pre- (skin/rib/chest wall) and post-focal (vertebra) regions of HIFU beams. HIFU resulted in the deterioration of liver function (3.77%), hydrothorax (2.59%), severe abdomen pain (1.77%), gastroenteric dysfunction (1.00%), cholecystitis (0.23%), cholangiectasis (0.23%), cardiac events (0.55%), hydropericardium (0.09%) and hematuria (0.36%). Serious AEs included tumor or vessel rupture, intrahepatic metastasis, lung embolism, renal failure and death.

#### Pancreas

150 AEs (11 types) were reported in 1717 patients (8.74%). Burns were reported in 3.09% of the cases. Pancreatitis (1.86%) and diabetes (1.28%) were the specific toxicities. HIFU occasionally led to bleeding, occlusion of the superior mesenteric artery and hepatic abscess.

#### Bone

9 kinds of complications were reported, with an overall rate of 20.54%. Frequent AEs were nerve injury (6.25%), skin burn (4.46%) and fracture (4.46%). Tumor rupture, epiphyseal separation and hemoglobinuria were detected in some patients.

#### Breast

Burns were the only reported AE, with a rate of 11.38%.

#### Soft tissues

The rate of AEs (burn, cutaneous necrosis and nerve injury) was 14.81%.

#### Prostate

5 types of AEs were reported. The rates of skin burn, hematuria, urinary obstruction, urethral stricture and incontinence were 7.20%, 17.33%, 2.40%, 1.07% and 0.53%, respectively.

**Figure 1:**
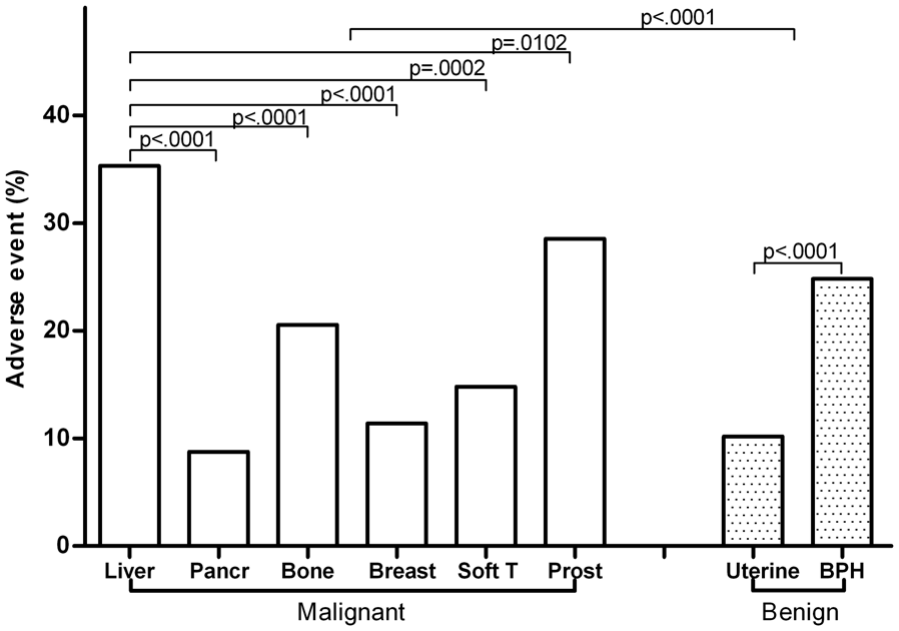
Comparison of AEs among 6 malignant and 2 benign disease types. Pancr: pancreas; Soft T: soft tissues; Uterine: uterine fibroid; Prost: prostate; BPH: benign prostate hyperplasia.

Rates of AEs differed among these 6 disease types (p<0.0001) and the highest rate occurred in liver cancer (Figure 1).

### AEs in benign diseases

AEs in the treatment of uterine fibroid or prostate hyperplasia were summarized in Table 2.

#### Uterine fibroid

563 AEs (8 types) were detected in 5526 patients (10.19%). The most frequent AEs were burn (2.44%), hematuria (2.88%), nerve injury (3.06%), and severe or prolonged abdominal pain (1.66%).

#### Prostate hyperplasia

The rate of AEs was 24.80%, including hematuria (17.78%), urinary irritation (4.30%) and urine retention (2.72%).

The rate of AEs in prostate hyperplasia was higher than that in uterine fibroid (p<0.0001) (Figure 1).

### AEs among the therapeutic devices

Of the 13262 cases, 6827 (51.48%) were treated with the device FEP-BY, 2896 (21.84%) with the device NIT-9000, 2296 (17.31%) with the device JC, 775 (5.84%) with the device 2001, 343 (2.59%) with the device CZ-901, 94 (0.71%) with the device 2000, and 31 (0.23%) with the device HY2900 (Table 1).

**Figure 2:**
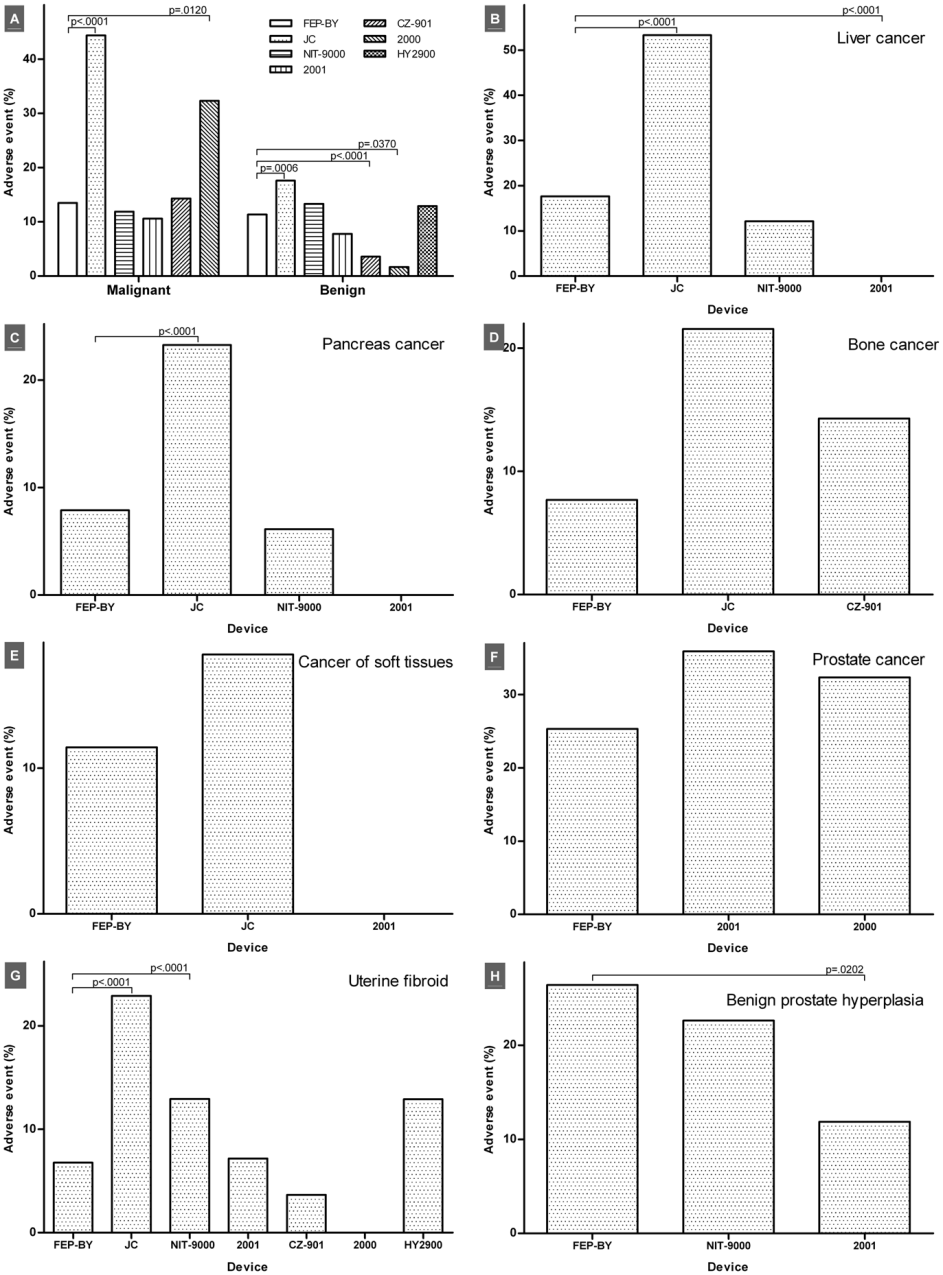
Comparison of AEs that occurred following treatment with different therapeutic devices. All diseases examined (*A*), cancers of liver (*B*), pancreas (*C*), bone (*D*), soft tissues (*E*) and prostate (*F*), uterine fibroid (*G*) and benign prostate hyperplasia (*H*).

Rates of AEs differed between therapeutic devices in both malignant (10.58–44.38%, p<0.0001) and benign diseases (1.67–17.57%, p<0.0001). Large variabilities in the disease type and the case number made it impossible to perform a comprehensive comparison. Uterine fibroid was the only disease treated with all of the HIFU devices, and the rate of AEs was 0–22.88% (p<0.0001). There were differences in the rates of AEs in cancers of liver (0–53.29%, p<0.0001) and pancreas (0–23.26%, p<0.0001) among the FEP-BY, JC, NIT-9000 and 2001 devices. The rates of AEs in prostate hyperplasia (11.86–26.40%, p = 0.0236) varied among the FEP-BY, NIT-9000 and 2001 devices. Rates of AEs were consistent in cancers of bone (7.69–21.57%, p = 0.3779), soft tissues (0–17.78%, p = 0.6191) and prostate (25.30–35.87%, p = 0.1448). In breast cancer, the JC device did not lead to more AEs compared with the NIT-9000 device (15.15% *vs.* 5.88%, p = 0.0542) (Figure 2A–H, Tables S1, S2, S3, S4, S5, S6, S7).

### Chronological incidence of AEs

**Figure 3:**
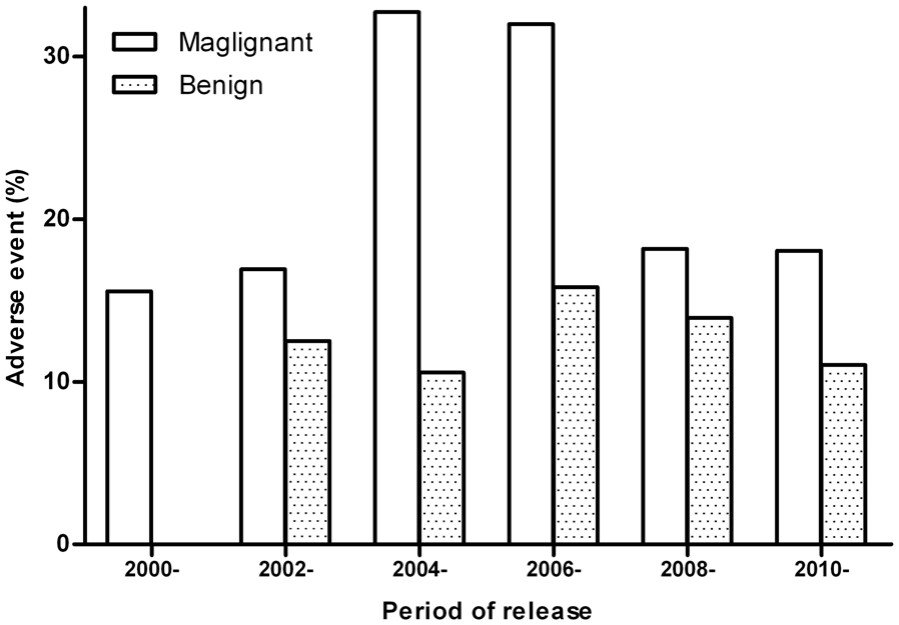
Chronological analysis of the rate of AEs. No trend over time towards a reduction in the rate was detected in either the malignant or benign disease types.

Rates of AEs were calculated chronologically. The date of HIFU treatment was not described in some articles, so the chronological analysis here was based upon the year of publication. Rates of AEs over time differed in the malignant disease, slightly varied in the benign disease, and no trend towards reduced rates over time was detected (Figure 3).

### Comparison with MRgFU for uterine fibroid and bone/breast cancer

USgFU treatment for uterine fibroid was compared with MRgFU treatment. Neurotoxicity was the AE that could be evaluated. Rates of neurological events were 7.34% in MRgFU and 4.72% (including severe abdomen pain) in USgFU (p = 0.2374) [12].

In the management of breast cancer, skin burn was detected in 1/30 patients treated with MRgFU, and the rate was 11.38% in USgFU treated patients (p = 0.3102) [4]. A multicenter trial did not demonstrate device-related side effects in the palliative treatment of bone metastasis with MRgFU [3]. The rate of AEs was 20.54% in treatment of bone cancer using USgFU (p = 0.0003), including burn, nerve injury, fracture, tumor rupture and death.

### Comparison with transrectal HIFU for prostate diseases

USgFU was compared with transrectal HIFU for prostate diseases. Rates of AEs were 28.53% in cancer and 24.80% in benign hyperplasia, which were consistent with those in transrectal HIFU [5], [13], [14]. Incidences of hematuria, the only comparable AE in benign hyperplasia, were 17.78% and 9.6% for the USgFU and transrectal HIFU treatments, respectively (p = 0.1757) [13]. However, the most frequent AEs following transrectal HIFU were urinary incontinence (6–27%) and erectile dysfunction (50–77%) in patients with prostate cancer and hematospermia (20%) in benign hyperplasia, these rates were not reported for USgFU treatment [13], [14].

## Discussion

### The location of the disease as a determinant of AEs

The types of AEs measured indicated that they were engendered by ultrasonic lesions. HIFU therapy requires the application of heat and cavitation, which may produce AEs [1], [2]. Burn and visceral perforation suggest thermal lesions. Tumor/vessel rupture or bleeding, ectopic embolism and intrahepatic spread result from cavitation. Cavitation detaches cancer cells/emboli from the primary site and thereby releases them into the circulation, leading to metastasis or embolism [15], [16]. Indeed, most of AEs may be related to the combination of heat and cavitation. Cavitation increases the sensitivity of tissue to heat, thereby extending lesions beyond the HIFU focus [17]. Severe events may be induced if vital structures are in the vicinity of the lesion.

The present data indicated that AEs frequently occurred in tissues adjacent to the target lesion and lying in the travel path of the HIFU beams (*i.e.*, the occurrence of untoward lesions depended on the location of the lesion). Thus, selecting a proper delivery path for the HIFU beams *in vivo* improves the safety profile of this technique. The energy in the post-focal region is lower than that in the pre-focal region, for ultrasound attenuates exponentially over distance traveled in tissues; thereby the post-focal region may be at a lower risk of developing AEs. However, the distribution of vertebra (in liver, pancreas and uterine tumors), kidney (in liver cancer) and nerve injuries (in uterine fibroid) demonstrated that ultrasonic lesions can occur in the post-focal regions. These injuries may be caused by the refocusing of ultrasound beams in tissues [18]. This refocusing may lead to lesions in distant tissues. For example, refocusing may cause arrhythmias or hydropericardium when the patient was being treated for liver cancer. The behavior of ultrasound in tissues is difficult to predict, which is further complicated by the heterogeneity of biological tissues. A longer travel distance of HIFU beams from the transducer to a target lesion indicates a more complex ultrasound-tissue interaction, thereby increasing the risk of inducing untoward effects.

Intrahepatic metastasis was observed in patients that were treated for liver cancer [19]. This is inconsistent with previous assertions that HIFU does not enhance cancer metastasis [1], [2]. The failure to detect unaffected cancerous tissues during HIFU treatment may play an important role. Tumor rupture, which sometimes occurred, may be involved in the formation of metastasis [20]. Rupture of esophageal varices after HIFU occurred in some cases [21], [22]. HIFU destroyed the shunt vessels, thereby increasing the risk of angiorrhexis by increasing the intravascular pressure. The delivery of abundant ultrasonic energy into the body over short periods of time led to a rapid rise of body temperature (up to 39.2°C), which overwhelmed the capacity of body to modulate heat. Ultrasound-induced overheating can be alleviated by suspending insonation or decreasing the intensity [23], [24]. Thus, body heat should be closely monitored when the treatment includes high intensities or longer exposure durations.

Diabetes was a serious toxicity when treating pancreas cancer with USgFU, with an incidence of 50% on occasion [19]. Surrounding tissues are usually covered during cancer treatment with HIFU [18]. Some islets are therefore destroyed directly. Moreover, the scattering of HIFU beams within the pancreas reduces the function of β cells, because a nonlethal level of insonation can modulate the cellular function [25]. These two factors lead to diabetes *via* decreasing the yield of insulin. Pancreatitis is mediated by similar mechanisms.

Peripheral nerve injuries have been observed following treatment of cancers of bone and soft tissues, which can be reversible or irreversible [26]. The prognosis depends on the type of lesion. Irreversible damage may result from focal lesions (including the refocusing of HIFU in tissues) when the nerve trunk lies in the focus of the HIFU beams. Much lower energy passes through the beams of ultrasound scattering, thereby inducing reversible damage. Hydrothorax, cholecystitis, and gastroenteric dysfunctions may also be due to HIFU scattering.

Ischiadic or sacral nerve damages and hematuria were the most frequent AEs following the treatment of uterine fibroid. Insonation harms the bladder, thereby inducing hematuria. Ischiadic and sacral nerves lie behind the focus of the HIFU beams, and their lesions are usually mediated by HIFU scattering and can recover in most cases [27].

The rate of AEs in malignant diseases was higher than that in benign ones. The reasons for this may include: (1) cancers usually require radical ablations (*i.e.*, destroying both the lesion and any tissue that is definitely adjacent to the lesion), thereby yielding a higher probability of inducing unexpected tissue damages [18]. Indeed, the deterioration of liver function in liver cancer, and the fracture and epiphyseal separation in bone cancer are related to the destruction of surrounding noncarcinous tissues; (2) chemotherapy or radiotherapy is usually undertaken perioperatively to improve the therapeutic efficacy even though it does not always augment HIFU effectiveness, which impairs noncarcinous tissues and thereby increases their sensitivity to HIFU scattering [18], [28]; (3) a lesion includes critical structures that cannot be avoided when directing the travel path of HIFU beams. This was observed in patients with occlusion of the superior mesenteric artery [29]. However, previous investigations indicate that a major vessel cannot be damaged by HIFU, because of heat transfer by blood flow [30]. Occlusion of an artery may result in necrosis of normal tissues supplied by this vessel, which must be considered.

Rates of AEs varied between disease types and a higher value (>20%) occurred following the treatment of liver, bone or prostate disease. As such, the location of a disease plays an important role. A lesion with more vulnerable structures in the vicinity has a higher incidence of untoward events, and more vital structures nearby suggest a higher risk of serious AEs. The pathological type of a disease may not be a critical determinant of AEs.

### The therapeutic device as a determinant of AEs

Theoretically, comparing AEs between treatments with different therapeutic devices should be conducted under identical HIFU intensity/frequency and insonation parameters. However, those parameters varied considerably in the literature, even for the treatment of a single disease type. HIFU works in the range of nonlinear acoustics, and biologic responses vary drastically between tissues types and individuals [31]. Accordingly, to ablate a volume completely, the intensity needs to be modulated constantly in HIFU exposure according to tissue responses [18]. These show that HIFU therapy is not a standardized procedure, with a low level of evidence from the perspective of evidence-based medicine, and that the therapeutic device is a determinant of the rate of AEs. However, control trials that explore the relationship between AEs and HIFU devices are difficult. The present data therefore should only be used as a reference for identifying the impact of HIFU device on AEs.

Because ultrasonography is a rapid imaging technique, it may be possible to monitor tissue responses in real time during USgFU treatment [2]. It is possible, therefore, that USgFU may decrease the rate of AEs compared with MRgFU. This hypothesis was not supported by the present data. USgFU was limited by the lower resolution of its ultrasonic images and the use of diagnostic ultrasounds with lower frequency (3.0–4.0 MHz; for observing deeper tissues). The specificity and negative predictive values were low when using ultrasonic images to predict tissue necrosis in real time (*i.e.*, sometimes destroyed tissues cannot be identified) [32]. This may result in longer insonation durations that allow for the induction of untoward tissue lesions. Tissues beyond the scope of the diagnostic ultrasound but in the propagation path of the therapeutic beams (*i.e.*, the blind field) are at a high risk of being harmed by HIFU [18]. This may contribute to ultrasonic lesions that were formed in ribs/chest wall in patients being treated for liver cancers.

AEs in USgFU were compared with those in MRgFU and transrectal HIFU. During treatments of uterine fibroid, AEs observed following USgFU were likely HIFU-related, but most AEs following MRgFU (discomfort, pain, and gynecologic/cardiovascular/respiratory symptoms) were likely not related to the treatment [12]. Urinary incontinence, erectile dysfunction, and hematospermia frequently occurred in patients being treated for prostate diseases with transrectal HIFU [13], [14]. Those AEs were not reported following USgFU, and the difference cannot be accounted for by the physical and/or medical profiles. A reasonable possibility was that those AEs were not monitored in the clinical trials using USgFU. AEs mentioned in those trials included in this study were almost events that occurred soon after the treatment, and later complications were unavailable in the published data. AEs resulted from USgFU treatment, therefore, remain to be thoroughly described.

USgFU is not a standardized therapeutic process. An optimal insonation regime depends on the experience of an operation team, thereby increasing the probability of over- or under-sonication. Over-sonication may extend the lesion to nontarget tissues, and under-sonication may result in residual intact tissues that facilitate cancer relapse and metastasis. Ultrasound should be delivered into the lesion in a few seconds to realize tissue ablation. A higher intensity favors the energy deposition within the target volume [18]. This also increases the intensity within tissues outside the focus increasing the risk of untoward lesions. A HIFU device employing a higher intensity therefore has a higher incidence of AEs–the highest rate of AEs occurred following treatments with the device JC in the present data. It is reasonable to expect that AEs may be reduced with the development of HIFU devices and greater clinical experiences. However, chronological analysis did not demonstrate a trend towards a reduction in the rates of AEs over time. USgFU modality, therefore, is still at an early stage.

### Limitations and summary

The case number varied drastically between disease types and between HIFU devices, and the disease types treated differed among HIFU devices. These limited a systemic evaluation of AEs; thus AEs can only be compared in some disease types between some HIFU devices in this study. The insonation parameter and mode were not described detailedly in literatures, so their impacts on AEs cannot be deduced. The safety of therapeutic modalities should be compared in a specific cohort. However, AEs of MRgFU or transrectal HIFU were from a Western population for lack of the data in a Chinese population, which was another limitation.

In summary, AEs following USgFU treatment were not yet thoroughly understood. Side effects were dependent upon the location of the lesion and the HIFU device used in its treatment. High incidences of AEs in some disease types indicated that the use of USgFU therapy should be curtailed in some cases. Indeed, USgFU therapy should be restricted to carefully selected cases. Rigid guidelines should be developed to calibrate and monitor the use of HIFU devices because AEs were related to the therapeutic device.

## Supporting Information

Table S1 Summary of AEs related to the use of the device FEB-BY.
(PDF)

Table S2 Summary of AEs related to the use of the device JC.
(PDF)

Table S3 Summary of AEs related to the use of the device NIT-9000.
(PDF)

Table S4 Summary of AEs related to the use of the device 2001.
(PDF)

Table S5 Summary of AEs related to the use of the device CZ-901.
(PDF)

Table S6 Summary of AEs related to the use of the device 2000.
(PDF)

Table S7 Summary of AEs related to the use of the device HY2900.
(PDF)
