# Supplementary material for: Adverse Events of Extracorporeal Ultrasound-Guided High Intensity Focused Ultrasound Therapy
Source: PLoS One. 2011 Dec 14;6(12):e26110. doi: 10.1371/journal.pone.0026110 (PMC3237413; doi:10.1371/journal.pone.0026110)
Supplement: Table S1 — Summary of AEs related to the use of the device FEB-BY. (PDF) [file pone.0026110.s001.pdf]

Table S1 Summary of AEs related to the use of the device FEB-BY

Disease	Case	Adverse event	Incidence
Malignant			
Liver	889	Skin burn 71<br>Rib injury 5<br>Palpitation 4<br>ALT/AST elevation 15<br>Jaundice aggravation 2<br>Gastroenteric dysfunction 7<br>Severe abdomen pain 39<br>Bleeding/liquefaction 7<br>Intrahepatic metastasis 7	17.66% (157/889)
Pancreas	1411	Burn 27<br>Jaundice aggravation 8<br>Diabetes 22<br>Pancreatitis 26<br>Steatorrhea 13<br>Gastroenteric dysfunction 9<br>Collapse 3<br>Bleeding 2<br>Hepatic abscess 1	7.87% (111/1411)
Bone	13	Skin burn 1	7.69% (1/13)
Soft tissues	35	Burn 4	11.43% (4/35)
Prostate	249	Skin burn 24<br>Hematuria 26<br>Urinary obstruction 9<br>Urethral stricture 2<br>Incontinence 2	25.30% (63/249)
Kidney	7		
Adrenal	18	Skin burn 2<br>Rib injury 5<br>Palpitation 2	50.00% (9/18)
Bladder	223	Hematuria 47<br>Urinary irritation 14<br>Spasm 1	27.80% (62/223)
Digestive tract	81	Skin burn 2	2.47% (2/81)
Metastatic nodes	28	Skin burn 27	96.43% (27/28)

Retroperitoneal metastasis	130	Skin burn 4	3.08% (4/130)
Celiac metastatic lesions	122	Skin burn 4<br>Severe pain 6<br>Gastroenteric dysfunction 12	18.03% (22/122)
Unspecified	747	Skin burn 20<br>Nerve injury 6<br>Hematuria 21<br>Intestinal obstruction 7<br>Gastroenteric dysfunction 6<br>Bleeding 6<br>Faint 5<br>Dysuria 1	9.64% (72/747)
	3953	534	13.51%
Benign			
Uterine fibroid/adenomyoma	2154	Skin burn 56<br>Nerve injury 59<br>Severe abdomen pain 2<br>Hematuria 22<br>Urinary irritation 1<br>Gastroenteric dysfunction 6	6.78% (146/2154)
Prostate hyperplasia	678	Hematuria 122<br>Urinary irritation 33<br>Urine retention 24	26.40% (179/678)
Secondary hyperparathyroidism	2	Skin burn 1	50% (1/2)
Hypersplenism	3		
Chyluria	20		
Ectopic pregnancy	16		
Dermoid cyst	1		
	2874	326	11.34%
Total	6827	860	12.60%
