# Supplementary material for: Adverse Events of Extracorporeal Ultrasound-Guided High Intensity Focused Ultrasound Therapy
Source: PLoS One. 2011 Dec 14;6(12):e26110. doi: 10.1371/journal.pone.0026110 (PMC3237413; doi:10.1371/journal.pone.0026110)
Supplement: Table S2 — Summary of AEs related to the use of the device JC. (PDF) [file pone.0026110.s002.pdf]

Table S2 Summary of AEs related to the use of the device JC

Disease	Case	Adverse event	Incidence
Malignant			
Liver	1156	Skin burn 420<br>Chest wall injury 2<br>Vertebra injury 4<br>Supraventricular tachycardia 8<br>Hydropericardium 2<br>Hypertension 8<br>Cholecystitis 5<br>Intrahepatic cholangiectasis 5<br>ALT/AST elevation 66<br>Gastroenteric dysfunction 15<br>Tumor rupture 2<br>Rupture of esophageal varices 1<br>Lung embolism 2<br>Hydrothorax 57<br>Pneumonedema 1<br>Asthma 2<br>Hematuria 8<br>Creatine elevation 2<br>Renal failure 1<br>High fever 4<br>Death 1	53.29%<br>(616/1156)
Pancreas	129	Skin burn 16<br>Vertebra burn 2<br>Amylase elevation 6<br>Jaundice aggravation 1<br>Gastrointestinal dysfunction 4<br>Occlusion of the superior mesenteric artery 1	23.26%<br>(30/129)
Bone	204	Skin burn 9<br>Nerve injury 14<br>Fracture 10<br>ALP elevation 5<br>Tumor rupture 1<br>Infection 2<br>Epiphyseal separation 1<br>Hemoglobinuria 1<br>Death 1	21.57%<br>(44/204)
Breast	99	Skin burn 15	15.15%<br>(15/99)

Soft tissues	45	Skin burn 1<br>Cutaneous necrosis 4<br>Nerve injury 3	17.78%<br>(8/45)
Kidney	4		
Chest/abdomen wall metastasis	41	Skin burn 16<br>Skin numb 3<br>Bowel perforation 1<br>Dyspnea 1	51.22%<br>(21/41)
Intraperitoneal recurrence/metastasis	15	Skin burn 15<br>Skin numb 5<br>Occlusion of the superior mesenteric artery 1<br>Bowel perforation 1<br>Gastrointestinal dysfunction 13<br>Hypertension 2<br>ALT/AST elevation 2<br>Creatine elevation 1<br>Hematuria 2	280%<br>(42/15)
Seminal vesicle	1	Skin burn 1	100%<br>(1/1)
Unspecified	84	Skin burn 7<br>Hydrothorax 3<br>Bowel perforation 2	14.29%<br>(12/84)
	1778	789	44.38%
Benign			
Uterine fibroid/ adenomyoma	389	Skin burn 27<br>Vertebra burn 23<br>Nerve injury 21<br>Severe abdomen pain 15<br>Hematuria 3	22.88%<br>(89/389)
Breast fibroadenoma	128		
Fibroma	1	Limb necrosis 1<br>Hemoglobinuria 1	200%<br>(2/1)
	518	91	17.57%
Total	2296	880	38.33%
