# Supplementary material for: Adverse Events of Extracorporeal Ultrasound-Guided High Intensity Focused Ultrasound Therapy
Source: PLoS One. 2011 Dec 14;6(12):e26110. doi: 10.1371/journal.pone.0026110 (PMC3237413; doi:10.1371/journal.pone.0026110)
Supplement: Table S3 — Summary of AEs related to the use of the device NIT-9000. (PDF) [file pone.0026110.s003.pdf]

Table S3 Summary of AEs related to the use of the device NIT-9000

Disease	Case	Adverse event	Incidence
Malignant			
Liver	33	Skin burn 2	12.12%
		Rib injury 2	(4/33)
Pancreas	147	Skin burn 8	6.12%
		Jaundice aggravation 1	(9/147)
Breast	68	Skin burn 4	5.88%
			(4/68)
Retroperitoneal metastasis	89	Burn 3	3.37%
			(3/89)
Unspecified	184	Burn 13	22.83%
		Nerve injury 28	(42/184)
		Hematuria 1	
	521	62	11.90%
Benign			
Uterine fibroid/adenomyoma	2190	Skin burn 12	12.92%
		Nerve injury 75	(283/2190)
		Severe abdomen pain 66	
		Hematuria 129	
		Hemafecia 1	
Prostate hyperplasia	146	Hematuria 28	22.60%
		Urinary irritation 5	(33/146)
Endometriosis	8		
Ectopic pregnancy	31		
	2375	316	13.31%
Total	2896	378	13.05%
