# Supplementary material for: Adverse Events of Extracorporeal Ultrasound-Guided High Intensity Focused Ultrasound Therapy
Source: PLoS One. 2011 Dec 14;6(12):e26110. doi: 10.1371/journal.pone.0026110 (PMC3237413; doi:10.1371/journal.pone.0026110)
Supplement: Table S4 — Summary of AEs related to the use of the device 2001. (PDF) [file pone.0026110.s004.pdf]

Table S4 Summary of AEs related to the use of the device 2001

Disease	Case	Adverse event	Incidence
Malignant			
Liver	123		
Pancreas	30		
Soft tissues	1		
Prostate	92	Skin burn 3<br>Hematuria 28<br>Urethral stricture 2	35.87%<br>(33/92)
Kidney	8		
Bladder	14		
Uterine cervix	6		
Bile duct	3		
Digestive tract	32		
Intraperitoneal metastasis	3		
	312	33	10.58%
Benign			
Uterine fibroid	404	Skin burn 10<br>Nerve injury 14<br>Hematuria 5	7.18%<br>(29/404)
Prostate hyperplasia	59	Hematuria 7	11.86%<br>(7/59)
	463	36	7.78%
Total	775	69	8.90%
