# Supplementary material for: Adverse Events of Extracorporeal Ultrasound-Guided High Intensity Focused Ultrasound Therapy
Source: PLoS One. 2011 Dec 14;6(12):e26110. doi: 10.1371/journal.pone.0026110 (PMC3237413; doi:10.1371/journal.pone.0026110)
Supplement: Table S5 — Summary of AEs related to the use of the device CZ-901. (PDF) [file pone.0026110.s005.pdf]

Table S5 Summary of AEs related to the use of the device CZ-901

Disease	Case	Adverse event	Incidence
Malignant			
Bone	7	Tumor rupture 1	14.29% (1/7)
	7	1	14.29
Benign			
Uterine fibroid	328	Burn 3<br>Severe/prolonged abdomen pain 9	3.66% (12/328)
Bone	8		
	336	12	3.57%
Total	343	13	3.79%
