# Supplementary material for: Adverse Events of Extracorporeal Ultrasound-Guided High Intensity Focused Ultrasound Therapy
Source: PLoS One. 2011 Dec 14;6(12):e26110. doi: 10.1371/journal.pone.0026110 (PMC3237413; doi:10.1371/journal.pone.0026110)
Supplement: Table S6 — Summary of AEs related to the use of the device 2000. (PDF) [file pone.0026110.s006.pdf]

Table S6 Summary of AEs related to the use of the device 2000

Disease	Case	Adverse event	Incidence
Malignant			
Prostate	34	Hematuria 11	32.35% (11/34)
	34	11	32.35%
Benign			
Uterine adenomyoma	30		
Hypersplenism	30	Skin burn 1	3.33% (1/30)
	60	1	1.67%
Total	94	12	12.77%
