# Supplementary material for: Adverse Events of Extracorporeal Ultrasound-Guided High Intensity Focused Ultrasound Therapy
Source: PLoS One. 2011 Dec 14;6(12):e26110. doi: 10.1371/journal.pone.0026110 (PMC3237413; doi:10.1371/journal.pone.0026110)
Supplement: Table S7 — Summary of AEs related to the use of the device HY2900. (PDF) [file pone.0026110.s007.pdf]

Table S7 Summary of AEs related to the use of the device HY2900

Disease	Case	Adverse event	Incidence
Uterine fibroid/adenomyoma	31	Burn 4	12.90% (4/31)
Total	31	4	12.90%
